# A Parallel

**Published:** 1862-05

**Authors:** 


**A PARALLEL.** — The history of all past times does not afford a single, solitary example of a man's repenting that he had had too much practical faith in the Christian religion, but multitudes that they had too little; so, no man who has lived a regular, temperate life to a good old age has ever professed a regret that he had not lived differently. And as the mistaken advocates of false religious systems have bitterly regretted their delusions in the searching ordeal of a dying hour, so, on the other hand, do the victims of animal appetites and propensities and unmatured notions, pertaining to human well-being, deplore the folly which led them into plausible, untested, untried ways of living healthfully, happily, and long. Therefore, not more surely will that man attain "immortality and eternal life" who walks in the "old paths" of love to God and love to man, practically carried out in every day of his pilgrimage toward the tomb, than that those who "use this world as not abusing it" and its good things, will find a sweet satisfaction in the same as long as they live. Hence, they are wisest who live in the temperate use and rational enjoyment of all the good things of this life.

